# Laser Microdissection of the Alveolar Duct Enables Single-Cell Genomic Analysis

**DOI:** 10.3389/fonc.2014.00260

**Published:** 2014-09-24

**Authors:** Robert D. Bennett, Alexandra B. Ysasi, Janeil M. Belle, Willi L. Wagner, Moritz A. Konerding, Paul C. Blainey, Saumyadipta Pyne, Steven J. Mentzer

**Affiliations:** ^1^Laboratory of Adaptive and Regenerative Biology, Brigham and Women’s Hospital, Harvard Medical School, Boston, MA, USA; ^2^Institute of Functional and Clinical Anatomy, University Medical Center of the Johannes Gutenberg-University, Mainz, Germany; ^3^Broad Institute of Massachusetts Institute of Technology, Harvard University, Cambridge, MA, USA; ^4^CR Rao Advanced Institute of Mathematics, Statistics and Computer Science, Hyderabad, India

**Keywords:** single-cell analysis, laser microdissection, microfluidics, alveolar duct, murine lung gene expression, signaling network, regenerative medicine

## Abstract

Complex tissues such as the lung are composed of structural hierarchies such as alveoli, alveolar ducts, and lobules. Some structural units, such as the alveolar duct, appear to participate in tissue repair as well as the development of bronchioalveolar carcinoma. Here, we demonstrate an approach to conduct laser microdissection of the lung alveolar duct for single-cell PCR analysis. Our approach involved three steps. (1) The initial preparation used mechanical sectioning of the lung tissue with sufficient thickness to encompass the structure of interest. In the case of the alveolar duct, the precision-cut lung slices were 200 μm thick; the slices were processed using near-physiologic conditions to preserve the state of viable cells. (2) The lung slices were examined by transmission light microscopy to target the alveolar duct. The air-filled lung was sufficiently accessible by light microscopy that counterstains or fluorescent labels were unnecessary to identify the alveolar duct. (3) The enzymatic and microfluidic isolation of single cells allowed for the harvest of as few as several thousand cells for PCR analysis. Microfluidics based arrays were used to measure the expression of selected marker genes in individual cells to characterize different cell populations. Preliminary work suggests the unique value of this approach to understand the intra- and intercellular interactions within the regenerating alveolar duct.

## Introduction

Complex tissues such as the lung are composed of structural hierarchies such as alveoli, alveolar ducts, and lobules (1). Recent evidence indicates that these structures reflect not only mechanical relationships, but also functional units that selectively participate in integrated processes such as tissue regeneration (2) and the development of bronchioalveolar carcinoma (3). Because bulk analyses of cells and gene expression – ignoring these regenerative units – have failed to illuminate the interactions between cells participating in these processes (4, 5), there is growing interest in the spatial sampling of the cells within regenerative units.

The initial attempts to refine the bulk analysis of cells within regenerating tissues used flow cytometry (FC) (6). FC is a multi-dimensional high-throughput technology that can analyze and sort individual cells based on their phenotypic characteristics – in most cases, sorting is based on cell surface molecule expression (7, 8). This approach has provided useful insights into the molecular expression of cell populations participating in lung regeneration (9–12); nonetheless, the relatively large number of cells sorted by FC has potentially obscured the cell–cell interactions within individual regenerative units.

To address these limitations, there is growing interest in isolating cells within individual regenerative units; in lung regeneration, the anatomic unit appears to be the alveolar duct. This goal has been aided by two developments. First, computer-controlled laser microdissection of tissue has facilitated the rapid isolation of small anatomic units within complex tissues. The ability to harvest anatomic units, such as the alveolar duct, under physiologic conditions has enabled detailed single-cell analysis. Second, microfluidic devices capable of capturing and handling single cells allowed for the analysis of the small number of cells comprising anatomic units such as the alveolar duct. Furthermore, these isolated cells can now be analyzed for gene expression at the level of individual cells. The result is an opportunity to define not only the signaling interactions between cells, but also the molecular interactions and signaling pathways within cells (9, 11, 13).

In this report, we demonstrate the use of laser microdissection to isolate cells in the murine lung alveolar duct and the microfluidic isolation and gene expression analysis of individual cells within this structural unit.

## Materials and Methods

### Animals

Male mice, 8–10-week-old wild type C57BL/6 (Jackson Laboratory, Bar Harbor, ME, USA) were anesthetized as previously described (14). The care of the animals was consistent with guidelines of the American Association for Accreditation of Laboratory Animal Care (Bethesda, MD, USA) and approved by our Institutional Animal Care and Use Committee.

### Anesthesia and intubation

The animals were anesthetized with an intraperitoneal injection of ketamine 100 mg/kg (Fort Dodge Animal Health, Fort Dodge, IA, USA) and xylazine 6 mg/kg (Phoenix Scientific, Inc., St. Joseph, MO, USA). The animals were intubated under direct visualization with a standard 20 g angiocatheter (BD Insyte, Sandy, UT, USA) (14) for subsequent agarose instillation.

### Corrosion casting and SEM

The right ventricular outflow tract was cannulated via right ventriculotomy with a 2 mm olive-tipped cannula (Acufirm 1428LL, Dreieich, Germany). The pulmonary vessels were perfused with 15–20 ml of 37°C saline followed by a buffered 2.5% glutaraldehyde solution (Sigma, St Louis, MO, USA) at pH 7.40. After casting of the pulmonary microcirculation with PU4ii (VasQtec, Zurich, Switzerland), diluted with 20% methylmethacrylate monomers (Sigma), and caustic digestion, the microvascular corrosion casts were imaged after coating with gold in an argon atmosphere with a Philips ESEM XL30 scanning electron microscope as previously described (13).

### Embedding medium

Agarose at 3% (w/v) was thoroughly mixed with distilled H_2_O and warmed to 37°C. The trachea, cannulated with a 20 g Angiocath (BD Insyte), was infused with the 37°C agarose using the lowest pressure necessary to inflate the peripheral lung (typically 20 cm H_2_O pressure). At total lung capacity, the trachea was clamped and the lung block was placed in 4°C saline and allowed to harden.

### Precision-cut lung slices

Sectioning was performed with the Leica VT1000 S vibrating blade microtome (Leica Biosystems, Nussloch, Germany) using stainless steel razor blades (Gillette, Boston, MA, USA). The microtome was operated at the following adjustable settings: knife angle, 5–7°; sectioning speed, 0.05–0.2 mm/s; oscillation frequency, 80–100 Hz; and oscillation amplitude, 0.6 mm. Most sections were 200 μm thick and mounted on a polyethylene naphthalate (PEN) membrane frame slide (Life Technologies, Carlsbad, CA, USA). The precision-cut lung slices were immersed in Dulbecco’s Modified Eagle’s Medium (DMEM) (Cellgro, Herndon, VA, USA) at 4°C prior to laser microdissection.

### Laser microdissection

The Arcturus XT LCM System (Life Technologies) was used for all ultraviolet (UV) laser dissection. The UV laser was a specially adapted beta-test laser for wet tissue applications. The enhanced UV cutting laser was a solid-state, diode-pumped (345 nm) laser with adjustable current (0–100%) and pulse frequency (10–5000 Hz). After mounting on the Arcturus stage, the surface of the lung slice was blotted dry. The alveolar ducts were optically targeted using a Nikon Eclipse Ti-E microscope with 10× and 20× Nikon CF objectives with high-intensity LED illumination. The stage was adjusted using a motorized trackball actuated in *X*- and *Y* -axes with 1 μm precision. The UV laser was empirically adjusted between 30 and 50% of maximal current to ensure complete penetration of the tissue slice. The Arcturus XT software was used to target tissue for UV dissection.

### Enzymatic digestion

Enzymatic digestion of the lung reflected a previously published protocol (15). Briefly, collagenase Type IV and DNase I (Sigma) was used to dissociate the tissue. The digestion was performed at 37°C under constant agitation until dissolved. The digest was filtered through 35 μm nylon mesh in preparation for microfluidic analysis.

### Cell viability

BAL-derived cells were counted using a Neubauer hemacytometer (Fisher, Pittsburgh, PA, USA). Dead cells were excluded by trypan blue (Sigma, St. Louis, MO, USA). Viability was further assessed using the calcein and ethidium live/dead assay (16) and measured by microfluorimetry (CytoFluor 4000, Applied Biosystems, Foster City, CA, USA) (17).

### C1 microfluidics chip

The C1 single-cell auto prep array integrated fluidic circuit (chip) is the microfluidic component of the Fluidigm C1™ Single-Cell Auto Prep System. The medium chip (10–17 μm cell size) was used to capture single cells (96 on one chip) obtained from the laser microdissection. The chip was used to perform single-cell PCR using a custom 96 gene panel designed for the alveolar duct.

### Flow cytometry

After standard enzymatic digestion of the lung (10), FC cell sorting using anti-CD31 mAb (APC or PE-Cy7; rat IgG2a, clone 390, eBioScience) (18) was used to isolate the CD31^+^ endothelial cells. Endothelial cell gene expression was assessed by PCR array as previously described (6).

### PCR gene expression

Comparison of single-cell and FC values were based on Ct values (6). For population analyses, the gene expression based on the ΔCt method (19) used an average of five housekeeping genes. The ΔCt values were normalized to an arbitrary 100-point linear scale for comparison with single-cell values.

## Results

### Precision-cut lung slices

**Figure 1:**
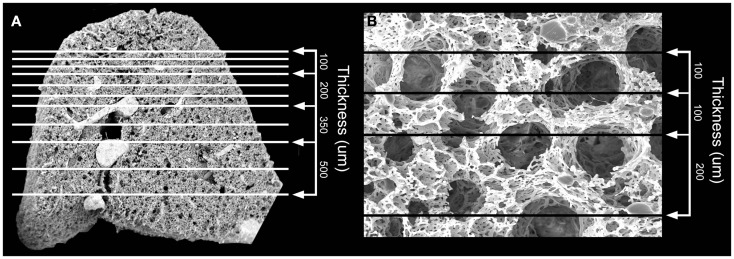
**Scanning electron microscopy (SEM) of the polymer casted murine lung**. **(A)** Cross-sectional 12x SEM of murine lung including representative size measurements at right. Vascular fixation and casting – the method with the least shrinkage of common lung preparation techniques – demonstrated bronchiolar and alveolar duct architecture when examined by SEM. **(B)** 200x SEM image of murine lung demonstrating size of alveolar ducts. The alveolar ducts were demonstrably contained within the 200 μm tissue slice.

**Figure 2:**
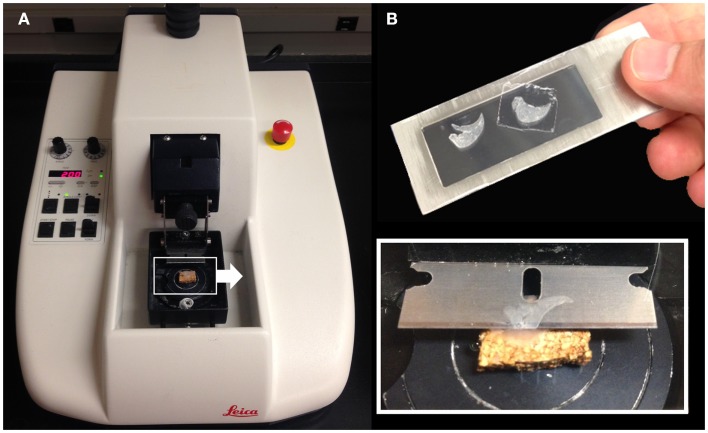
**Precision-cut lung slices were obtained by a vibrating blade microtome after agarose embedding (A)**. The agarose-lung block was initially mounted on a cork specimen platform with cyanoacrylate prior to sectioning (inset). The cut sections were subsequently mounted on a frame slide with a PEN membrane used for standard laser capture microdissection **(B)**.

**Figure 3:**
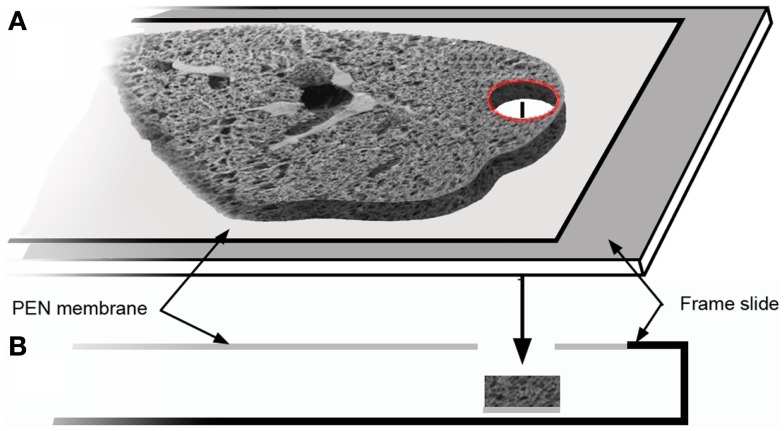
**The tissue slice was mounted on a frame slide**. The slide is composed of a standard optical microscope slide with a metal frame that supports a PEN membrane to facilitate laser microdissection **(A)**. In cross-section **(B)**, the frame slide creates a reservoir for retrieval of the dissected tissue.

Structural analysis of the murine lung using SEM demonstrated a mean diameter of the intact alveolar duct of 177 ± 31 μm (Figure 1). To obtain thickness-calibrated lung slices encompassing the alveolar ducts, 200 μm thick precision-cut lung slices were sectioned after intra-airway instillation of a 3% (w/v) agarose embedding medium. At 37°C, low melting point agarose had sufficiently low viscosity to permit instillation into the peripheral airspaces – important for the preservation of alveolar architecture. When cooled to 4°C, the agarose-lung block demonstrated stability for precision sectioning. The agarose-lung block, mounted on Leica VT1000 S specimen holder and immersed in ice-cold PBS, was serial sectioned (Figure 2). For single-cell genomics studies, the lung slices were mounted on a PEN membrane frame slide to provide structural support during laser dissection. The frame slide facilitated the retrieval and subsequent processing of the dissected specimen (Figure 3).

### Optical targeting

**Figure 4:**
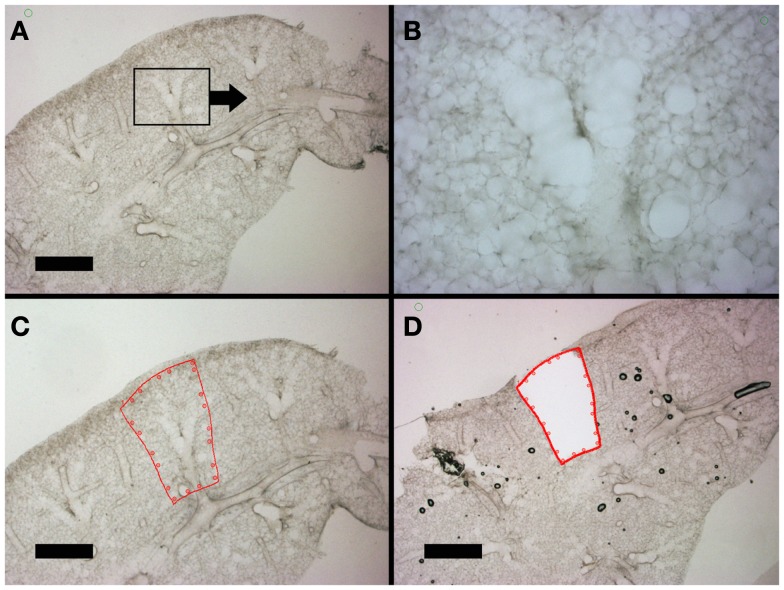
**Lung slices of the murine cardiac lobe examined by light microscopy**. After mounting on a frame slide, the sections were visualized without counter-stain at 10× **(A,C,D)** and 20× **(B)** magnification. The respiratory bronchiole and alveolar ducts were readily identified **(B)**. The relevant anatomic structure was demarcated by software annotation **(C)** and laser microdissected using computer control **(D)**. Successful dissection is demonstrated as the structure drops into the frame slide and out of the optical plane **(D)**. Bar = 1 mm.

The hierarchical anatomic structure of the lung facilitated the visual identification of the alveolar duct. The dehydrated lung used in laser capture loses structural detail when examined by light microscopy (not shown). In contrast, the well-hydrated lung used in laser microdissection – in the absence of counterstains or cell-specific labels – retains structural detail throughout the 200–300 μm thick precision-cut lung slices (Figure 4). Preliminary identification of the alveolar duct was confirmed by varying the optical plane and identifying the feeding bronchiole (Figures 4A,B). The computer-controlled laser software allowed for precise selection of regions encompassing the alveolar duct; the demarcated regions were harvested by the UV cutting laser (Figures 4C,D). The laser power, speed, and focal point were varied to optimize single-pass dissection of the tissue and minimize thermal damage. The required laser power was consistently reduced by 50% with absorbent blotting of the section surface followed by re-application of media immediately following UV dissection.

### Single-cell isolation

**Figure 5:**
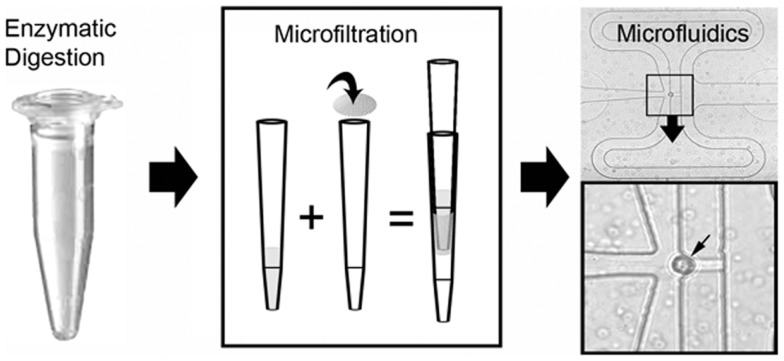
**Schematic of the single-cell isolation procedure after laser microdissection**. The tissue samples were enzymatically digested and microfiltered using a 35 μm mesh. Single cells were isolated and PCR performed on a C1 microfluidics chip. The successful capture of individual cells was readily identified by light microscopy (inset, small arrow).

The laser-dissected samples were enzymatically digested, microfiltered, and prepared for microfluidic analysis (Figure 5). The PEN membrane-associated samples were incubated in collagenase Type IV buffer for 15–30 min at 37°C with constant agitation. The acellular matrix and PEN membrane-associated debris were microfiltered with a 35 μm mesh. Routine examination of the filtered debris demonstrated virtually no residual cells. Cell yields varied from 1 to 3 × 10^3^ cells per alveolar duct. Cell viabilities, assessed by vital dye exclusion as well as live/dead assay microfluorimetry, ranged from 70 to 95% depending on treatment conditions. Live/dead confocal microscopy demonstrated dead cells at the laser-dissected sample margins (not shown), but cell viability from laser-dissected tissue (87 ± 5) was not statistically different from enzymatic digestion alone (95 ± 4) (*p* > 0.05). The cells were analyzed using the Fluidigm C1™ Single-Cell Auto Prep System (Fluidigm, South San Francisco, CA, USA). In the 96-well C1 system, a typical result was the capture of 75 single cells, 13 wells with 2 or more cells, and 2 wells with no cells.

### Single-cell PCR

**Figure 6:**
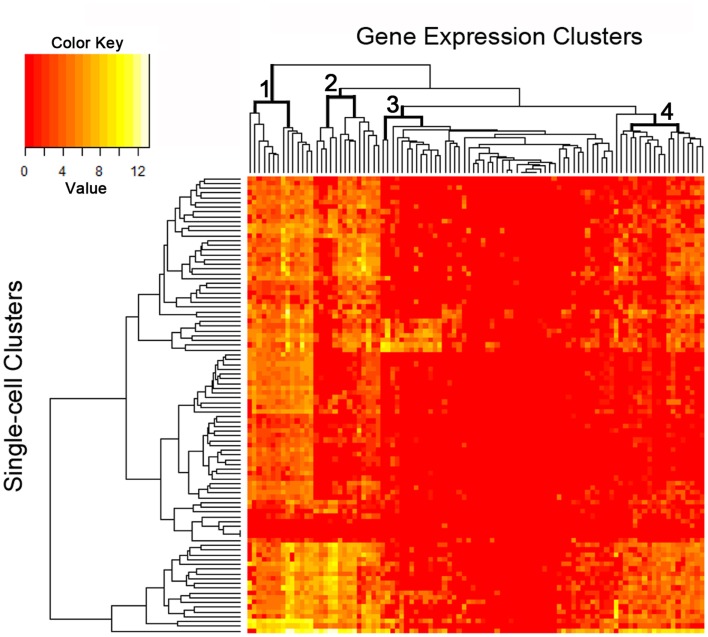
**The single-cell analysis data from the custom PCR panel processed using an unsupervised hierarchical clustering algorithm**. Phenotypic “markers” were incorporated into the gene expression panel to facilitate cell subset identification as well as correlation with histologic and flow cytometric studies. Four groups of markers are significantly expressed at single-cell level as shown in the figure (see top dendrogram).

**Figure 7:**
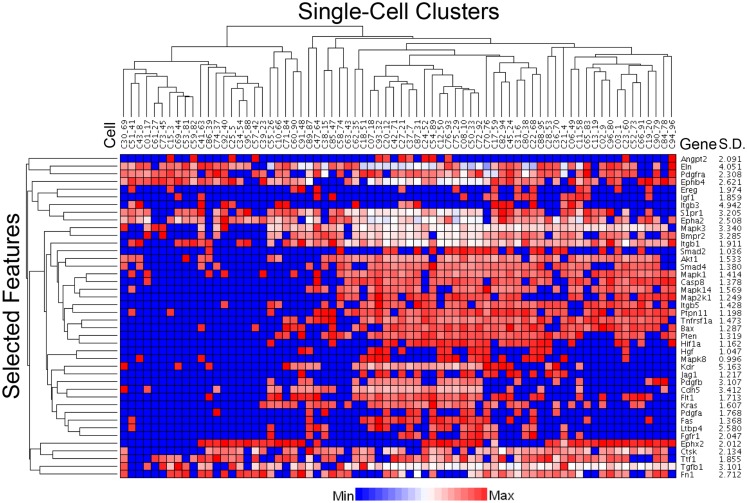
**Statistical processing of multiplexed samples to reduce noise and highlight selected features**. Upon filtering based on array QC readouts, and eliminating the low variation markers (SD less than 1), we performed feature selection based sparse hierarchical clustering of 72 single-cells. The clustering result, based on the selected features (42 marker genes), is shown. The marker-specific variation is indicated by standard deviation (Gene SD).

**Figure 8:**
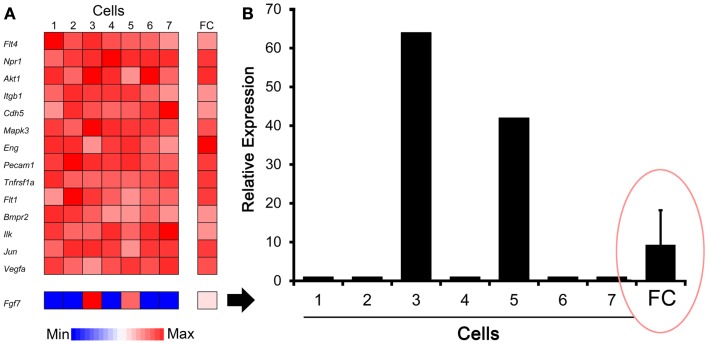
**Comparison of gene expression using single-cell analysis and flow cytometry (FC) cell isolation**. **(A)** Defined by endothelial cell marker genes (e.g., *Pecam1*), cells #1–7 expressed genes typically associated with endothelial cells. Some genes, such as *Fgf7* shown here, were expressed by only a subset of cells in the single-cell analysis. **(B)** Single-cell expression of the *Fgf7* gene was compared with seven single cells and FC sorted cell populations. The endothelial cells from normal lungs were isolated by FC cell sorting (10,000 cells based on CD31^+^ surface expression) as previously described (10). The results demonstrated population-averaged level of *Fgf7* expression that was not representative of any individual cell (ellipse). The gene expression was normalized to an arbitrary 100-point scale for comparison; error bar reflects 1 SD of triplicate samples.

The C1 chip used a microfluidic circuit to capture the isolated single cells (96 on one chip). The single-cell poly-adenylated RNA was converted to full-length cDNA for universal amplification of the cDNA and custom PCR analysis. Our custom PCR arrays were developed based on gene expression studies using FC-based phenotypic isolation of lung cells (6, 10–12, 20). Results of the C1 PCR studies were analyzed by unsupervised hierarchical clustering (Figure 6). Upon filtering based on array QC readouts, data on 72 single-cells were available. In addition, we have eliminated the genes that showed low variation (SD less than 1) followed by feature selection. Thus, we obtained a smaller matrix of 72 single-cells × 42 marker genes. Clustering based on selected features (using the Sparse Hierarchical Clustering module in GenePattern software from Broad Institute of MIT) demonstrated single-cell clusters (Figure 7) that corresponded to previously identified cell types (e.g., endothelial cells, myofibroblasts, alveolar macrophages, and alveolar epithelial cells). The information content of single-cell analysis was apparent when gene expression of endothelial cells (*Pecam1* gene expression) isolated by the C1 chip was compared to gene expression of endothelial cells (CD31^+^ surface expression) isolated by FC. Although the expression of many genes was similar in both groups (Figure 8A), some genes demonstrated variable expression patterns that were not reflected in the population-based FC analysis (Figure 8B).

## Discussion

In this report, we demonstrated laser microdissection of the lung alveolar duct for single-cell PCR analysis. Our approach involved three steps. (1) The initial preparation used mechanical sectioning of the lung tissue with sufficient thickness to encompass the structure of interest. In the case of the alveolar duct, the precision-cut lung slices were 200 μm thick and processed using near-physiologic conditions to preserve cell viability. (2) The lung slices were examined by transmission light microscopy to target the alveolar duct. The air-filled lung was sufficiently accessible by light microscopy that counterstains or fluorescent labels were unnecessary to identify the alveolar duct. (3) The enzymatic and microfluidic isolation of single cells for PCR allowed for the harvest of as few as several thousand cells for analysis. The data suggest the unique value of this approach to understanding the intra- and intercellular interactions within the regenerating or neoplastic alveolar duct.

Harvesting viable cells from the alveolar duct requires that tissue slices of the appropriate thickness have to be obtained in near-physiologic conditions. Manually sliced tissues have been used for nearly 90 years in various *in vitro* applications (21, 22); however, the manually prepared slices have had limited reproducibility and viability (23). A mechanical tool to facilitate the cutting of tissue of precise thickness was introduced by the Krumdieck in 1980 (24, 25). With progressive refinement, tissue microtomes now vibrate with varying amplitudes and frequencies to produce slices with highly reproducible thickness – thus, these sections are referred to as precision-cut tissue slices (26). With current vibrating microtomes, precision-cut tissue slices preserve the original organ architecture, the cellular heterogeneity, and extracellular matrix found *in vivo* (27). Precision-cut tissue slices, for example, have enabled the development of *ex vivo* systems for systematic pharmaco-kinetic profiling of tumors contained in their native 3-dimensional micro-environment (28).

A key feature of precision-cut lung slices in the lung is the use of agarose as structural support. Because live tissue is too soft for precise sectioning, and molten paraffin is too hot for live tissue, a biocompatible liquid with a low-temperature gelling point is used as an embedding medium. Agarose is typically used for lung embedding because it has sufficiently low viscosity at 37–40°C to be infused into the distal airspaces, yet is sufficiently rigid for microtome sectioning at 4°C.

Although there is a significant overlap between techniques, laser microdissection can be distinguished from laser capture microdissection (LCM) by the method of cell isolation. In the original LCM system developed at the National Institutes of Health, the target cells were identified by light microscopy and captured using an infrared laser (29). A transfer film positioned over the region of interest (typically tumor cells) was melted into the tissue using an IR laser. The dissected tissue microsample was then lifted from the histopathology slide for subsequent RNA, DNA, or protein analysis. In most contemporary LCM systems, a requirement for effective capture is the dehydration of the tissue – a requirement that precludes the study of live cells (30). In contrast, our laser microdissection approach uses a cutting UV laser to define the margins of the dissection under near-physiologic conditions. Although UV-induced thermal injury to the neighboring cells is demonstrable using live-dead assays; the dissection margins can be drawn to minimize the injury to the region of interest. In our application, we maintained near-physiologic conditions during the UV laser dissection with only blotting of the tissue surface to minimize excess liquid media prior to UV dissection. The UV laser-defined alveolar duct was subsequently transferred to the collection chamber without the use of an IR laser or transfer film; in most cases, the sample dropped into the chamber occasionally assisted by the application of liquid media.

A frequently under-appreciated characteristic of the lung is the large amount of extracellular matrix in the organ (31). In addition, some extracellular matrix components – most notably, elastin – are resistant to common enzymatic treatments (32). In our application, we have used a variety of enzymatic cocktails based on collagenase to facilitate dissolution of the laser-dissected tissue; however, the most important component has proven to be collagenase Type IV. In our protocols, we used 35-μm mesh to separate cells from the collagenase-resistant matrix debris. Routine re-examination of the filtered debris did not show residual cells suggesting that this approach does not result in the systematic loss of any particular cell subpopulation. Of note, cell filtration with mesh larger than 35 μm was prone to debris-induced occlusion of the microfluidics chip.

The cells isolated by microfluidics were studied using a custom 96 gene PCR panel. The results of prior FC population studies were used to select the genes used in this custom panel (6, 9–12, 20). FC-derived cell type-specific markers were incorporated into the panel to facilitate the spatial reconstruction of intercellular interactions within the alveolar duct. For example, the genes *Acta2* and *Pecam1* were added to the PCR panel to identify myofibroblasts and endothelial cells, respectively. Although the single-cell transcriptional data must be analyzed in the context of potential limitations, such as burst transcription (33, 34), insights derived from these data should be useful for re-interpreting population-based data both *in vivo* and *in vitro*.

Single-cell analysis is particularly relevant to the process of carcinogenesis within the alveolar duct. Bronchioalveolar stem cells, in addition to contributing to the maintenance and repair of the normal alveolar duct, have been shown to give rise to lung adenocarcinomas (35). The experimental challenge is that multipotent stem cells, like those giving rise to bronchioalveolar carcinoma, exist in low frequency within the peripheral lung. Population-based analyses are prone to overlook these cells and their transcriptional profile because of the “averaging” effect demonstrated in our study. Because of this limitation, we anticipate that single-cell analysis of the regenerating alveolar duct will provide important insights into the biology of bronchioalveolar stem cells.

Finally, these results demonstrate the ability to extract viable cells from a morphologically defined micro-region of a tissue sample. We successfully applied single-cell whole-transcriptome amplification and gene expression analyses to these samples – a process that allows for simultaneous region-specific and cell type-specific expression analysis. We anticipate even more interesting experiments are possible in the future. For example, similarly obtained viable single cells can facilitate a variety of experimental perturbations prior to endpoint analyses (e.g., expression profiling, nucleic acid sequencing, immunoassays, or proteomics). Furthermore, new statistical and bioinformatics techniques may be used to design efficient strategies for sampling cells at different locations in the tissue and at different time-points, and to study the genomics and transcriptomics of these cellular samples, thus leading to construction of spatio-temporal cell signaling networks. We are confident that such studies involving single-cell analysis will lead to a more nuanced understanding of the process of tissue regeneration.

## Conflict of Interest Statement

The authors declare that the research was conducted in the absence of any commercial or financial relationships that could be construed as a potential conflict of interest.

## Supplementary Material

The Supplementary Material for this article can be found online at http://www.frontiersin.org/Journal/10.3389/fonc.2014.00260/abstract

## Abbreviations

2D, 2-dimensional; 3D, 3-dimensional; IR, infrared; LCM, laser capture microdissection; PCR, polymerase chain reaction; PEN, polyethylene naphtalene; SEM, scanning electron microscopy; UV, ultraviolet; SCA, single-cell analysis; SD, standard deviation.
